# Cognitive bias modification of interpretations for anxiety and depression in children and adolescents: A meta‐analysis

**DOI:** 10.1002/jcv2.12207

**Published:** 2023-11-16

**Authors:** Gemma Sicouri, Emily K. Daniel, Michael J. Spoelma, Elske Salemink, Emma A. McDermott, Jennifer L. Hudson

**Affiliations:** ^1^ Black Dog Institute University of New South Wales Sydney New South Wales Australia; ^2^ School of Psychology University of New South Wales Sydney New South Wales Australia; ^3^ Discipline of Psychiatry and Mental Health School of Clinical Medicine Faculty of Medicine and Health University of New South Wales Sydney New South Wales Australia; ^4^ Department of Clinical Psychology Utrecht University Utrecht the Netherlands

**Keywords:** adolescent, anxiety, children, depression, interpretation bias modification

## Abstract

**Background:**

Evidence suggests that cognitive bias modification of interpretations (CBM‐I) is effective in modifying interpretation biases and has a small effect on reducing anxiety in children and adolescents. However, most evidence to date is based on studies which report anxiety or general distress using *ad‐hoc* Likert‐type or Visual Analogue Scales, which are useful but do not reliably index symptoms of clinical importance. This meta‐analysis aimed to establish the effects of CBM‐I for children and adolescents on both anxiety and depression using psychometrically validated symptom measures, as well as state negative affect and negative and positive interpretation bias.

**Methods:**

We identified studies through a systematic search. To be eligible for inclusion, studies needed to target interpretation biases, not combine CBM‐I with another intervention, randomly allocate participants to CBM‐I or a control condition, assess a mental health outcome (i.e., anxiety or depression symptoms using validated measures or state measures of negative affect) and/or interpretation bias and have a mean age less than 18 years.

**Results:**

We identified 36 studies for inclusion in the meta‐analysis. CBM‐I had a small and non‐significant unadjusted effect on anxiety symptoms (*g* = 0.16), no effect on depression symptoms (*g* = −0.03), and small and non‐significant unadjusted effects on state negative affect both at post‐training (*g* = 0.16) and following a stressor task (*g* = 0.23). In line with previous findings, CBM‐I had moderate to large unadjusted effects on negative and positive interpretations (*g* = 0.78 and *g* = 0.52). No significant moderators were identified.

**Conclusions:**

CBM‐I is effective at modifying interpretation bias, however there were no effects on mental health outcomes. The substantial variability across studies and paucity of studies using validated symptom measures highlight the need to establish randomized controlled trial protocols that evaluate CBM‐I in clinical youth samples to determine its future as a clinical intervention.

## Graphical abstract

Previous reviews evaluating CBM‐I in youth have found a small effect on anxiety outcomes, but little is known about the effect on anxiety and depression outcomes using validated symptom measures only, which would provide a better indicator of CBM‐I's clinical relevance. This meta‐analysis found CBM‐I had a small and non‐significant unadjusted effect on anxiety and depressive symptoms using validated measures, as well as state negative affect at post‐training and following a stressor. CBM‐I had moderate to large unadjusted effects on negative and positive interpretations. There were no significant moderators.

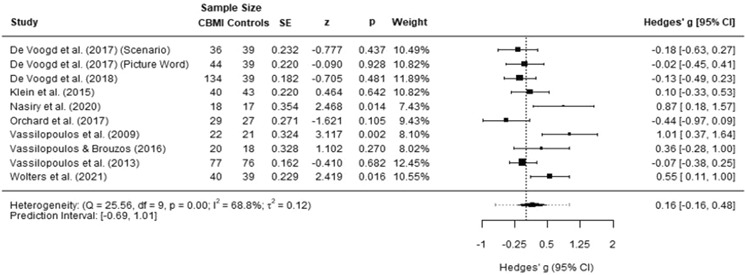

**Key points**

- Previous reviews evaluating cognitive bias modification of interpretations (CBM‐I) in youth have found a small effect on anxiety outcomes, but little is known about the effect on anxiety and depression symptoms using validated measures only, which would provide a better indicator of CBM‐I's clinical relevance.
- We conducted a meta‐analysis of between‐group effect sizes of CBM‐I on anxiety and depression symptoms using validated measures, negative affect using ad hoc or Likert scales, negative and positive interpretation bias.
- CBM‐I had a small and non‐significant unadjusted effect on all mental health outcomes. CBM‐I had moderate to large unadjusted effects on negative and positive interpretations. There were no significant moderators.
- Randomized controlled trials that evaluate CBM‐I in clinical youth samples using validated symptom measures are needed to determine if CBM‐I has a future as a clinical intervention.

## INTRODUCTION

Anxiety and depression are the most common and disabling mental health disorders in children and adolescents (Polanczyk et al., 2015). Cognitive behavioural therapy (CBT) is an evidence‐based treatment for both disorders, however up to 50% of children do not experience diagnostic remission (James et al., 2020; Yang et al., 2017). Access to evidence‐based care is also problematic, with only 19% of children and adolescents with these disorders accessing such care (Gandhi et al., 2022). There is a need to improve treatment access and treatment outcomes for children and adolescents with anxiety and depression by developing novel interventions. Cognitive bias modification of interpretations (CBM‐I) has been suggested as a potential solution.

CBM‐I uses repetitive training to encourage participants to endorse more positive or benign ways of thinking about ambiguous information over a threatening and negative interpretation to reduce associated anxious and depressive symptomatology. Training typically involves individuals being encouraged to resolve ambiguous scenarios in a more positive or benign way. CBM‐I has the potential to overcome many of the barriers that inhibit help‐seeking in children and young people, such as being delivered in a digital format and consisting of brief sessions requiring minimal therapist input. This makes it particularly attractive for treating anxiety and depression in youth as it is potentially an accessible and low‐cost treatment format compared to face‐to‐face CBT and could be offered during a waitlist or as an adjunct treatment.

CBM‐I was initially developed to assess the causal mechanisms between interpretation bias and anxiety and mood in healthy adult samples with varying levels of mental health symptoms (Mathews & Mackintosh, 2000). More recently, there is interest in its potential as a clinical tool (Blackwell, 2020). Some researchers have argued that CBM‐I has the potential to be particularly effective in youth samples because cognitive processing styles targeted in CBM‐I are still developing and may be more amenable to change (Haller et al., 2015; Lau et al., 2011). However, two meta‐analytic reviews have found mixed evidence for the efficacy of CBM‐I in children and adolescents for mental health outcomes. This is despite moderate to large effects on reducing negative bias and improving positive bias (Cristea et al., 2015; Krebs et al., 2017).

The first meta‐analysis, by Cristea et al. (2015), examined the efficacy of CBM‐I, in combination with attention bias modification (ABM), on bias and mental health outcomes. Mental health outcomes included all outcomes related to mental health problems, including general distress, anxiety, and depression. Relative to control or neutral training, CBM‐I (*k* = 13) had a moderate effect on bias outcomes (*g* = 0.52) and a small but non‐significant effect on mental health outcomes (*g* = 0.11). A small but significant effect size for mental health outcomes arose when interventions were delivered in school settings, but no other moderators were found to be significant. A second meta‐analysis by Krebs et al. (2017) examined the efficacy of CBM‐I on bias and anxiety outcomes only. Across 26 studies they found that CBM‐I had a moderate effect on negative and positive interpretations (*g* = 0.70 and 0.52 respectively). They also found a small effect on anxiety outcomes post‐training (*g* = 0.17) and following a stressor task (*g* = 0.34). No statistical moderators were found. These results provide encouraging—albeit tentative—evidence of the potential clinical utility of CBM‐I for anxious youth.

While these outcomes are encouraging, a limitation of these two reviews was that they pooled anxiety and general distress outcomes from studies that used both validated symptom measures and state outcomes using *ad‐hoc* Likert‐type or Visual Analogue Scales. While state measures are useful, they do not reliably index symptoms of clinical importance. A recent meta‐analysis evaluating cognitive bias modification interventions (including CBM‐I) in adults highlighted the need to move towards evaluating outcomes using symptom measures which are more relevant for clinical practice (Fodor et al., 2020). The authors of the two previous CBM‐I meta‐analyses in youth (Cristea et al., 2015; Krebs et al., 2017) acknowledged this limitation but chose to pool studies to increase statistical power. A greater number of CBM‐I studies have been conducted since these reviews and so an updated meta‐analysis which separates mental health outcomes using symptom measures and state measures is needed.

In addition, the reviews by Cristea et al. (2015) and Krebs et al. (2017) did not evaluate the effects of CBM‐I on depression outcomes. Given the high comorbidity between anxiety and depression in young people (Costello et al., 2003; Cummings et al., 2014), it is important to establish whether CBM‐I is effective as a transdiagnostic (i.e., across both anxiety and depression) or symptom‐specific intervention. Meta‐analytic findings in adults have found CBM‐I to have a similar effect on anxiety and depression symptoms using validated measures (Fodor et al., 2020), so it would be helpful to know whether CBM‐I has clinical utility for improving both anxiety and depressive symptoms in younger samples.

The primary aim of this study was to extend previous meta‐analyses evaluating CBM‐I in youth to determine whether CBM‐I modifies anxiety and depressive symptoms using validated and standardised measures, and measures of state negative affect. For state negative affect, we examined outcomes following CBM‐I training and following exposure to a challenging or stressful experience in accordance with diathesis‐stress models (MacLeod et al., 2004). These models indicate that cognitive biases are latent vulnerabilities that only exert an influence on states when the individual encounters a stressor. We also aimed to evaluate the extent to which CBM‐I improves negative and positive interpretation bias. The second aim was to explore the influence of potential moderators of outcomes. We selected moderators a priori that have been evaluated in the two prior meta‐analyses and which we expected to be associated with the effect of CBM‐I (Cristea et al., 2015; Krebs et al., 2017). Moderator variables were participant characteristics (clinical status, age and gender) and methodological characteristics (type of control group, number of training sessions).

## METHODS

This manuscript was developed in accordance with The PRISMA (Preferred Reporting Items for Systematic Reviews and Meta‐Analysis) Statement: An Updated Guideline for Reporting Systematic Reviews (Page et al., 2020) and was pre‐registered (Prospero: CRD42021254753).

### Literature search

We conducted a comprehensive literature search in PsycINFO, Ovid MEDLINE, PsycArticles, Web of Science, and EMBASE (Excerpta Medica DataBASE) using the terms: “interpret* bias AND training”; “interpret* bias AND modif*”; “cognitive bias AND training”; and “cognitive bias AND modif*” for publications between January 1900 and June 2023. Searches were limited to human populations aged below 18 years and English language studies. We also conducted searches of clinicaltrials.gov, the International Clinical Trials Registry Platform, and the Australian New Zealand Clinical Trials Registry using the search terms listed above, adapted for the conventions of each website. Finally, the reference sections of previous meta‐analyses were also searched for potentially relevant studies.

### Selection criteria

Studies meeting the following criteria were included: (a) randomised controlled trials; (b) comparing the effect of a CBM‐I intervention to a control condition; (c) where the CBM‐I training was delivered in isolation and not in combination with other interventions (e.g., ABM); (d) the control condition was negative CBM‐I training, neutral CBM‐I training, or no training/waitlist; (e) the study assessed anxiety symptoms (including obsessive compulsive disorder symptoms), depressive symptoms, state negative affect (following CBM‐I training and/or a stressor task), or interpretation bias; (f) participants were children or adolescents with a mean age equal to or less than 18 years; (g) data had not been previously reported in another paper eligible for inclusion; and (h) the study was published in a peer‐reviewed publication. Covidence was used to aid with the study selection process. All screening and full‐text review was conducted by two of the authors (EKD and MJS).

### Data extraction

We collected data on six outcome measures: (a) anxiety symptoms post‐training; (b) depressive symptoms post‐training; (c) state negative affect post‐training; (d) state negative affect post‐stressor task; (e) negative interpretation bias post‐training; and (f) positive interpretation bias post‐training. Outcomes were classified as anxiety or depressive symptoms if they used a validated symptom measure. Outcomes were classified as state negative affect if they used a state anxiety or mood measure, or a measure of general distress (e.g., nervous, sad, angry) on either an *ad‐hoc* Likert scale, Visual Analogue Scale, or a validated state measure (e.g., STAI‐C). We extracted raw score means, standard deviations and sample sizes for each group. For studies where raw means and standard deviations were not available, effect sizes or t‐values were extracted. We contacted authors for data in instances when studies did not report data needed to calculate an effect size. Authors of 15 studies were contacted and eight were responsive.

For moderator analyses, we extracted the following categorical variables: clinical status of the sample, age, type of control condition, and number of training sessions. Clinical status was divided in to diagnosed or high symptom (i.e., above a clinical cut off score) versus healthy samples. Age of participants was categorised as adolescent if the mean age was equal to or greater than 13 years, or child if it was below 13 years. For type of control condition, studies were divided into either a negative control (i.e., training in favour of a negative interpretation), neutral control (training in favour of positive or neutral interpretations for half of the trial and negative interpretations for the other half; or ambiguous scenarios that have no emotional valence), or no training/waitlist control groups. Number of sessions was divided into single session, two to four sessions, or greater than four sessions. Continuous moderators were age (years), gender (% female) and number of training sessions. Data extraction was conducted by one coder (EKD) and a random sample of 20% were checked by another coder (MJS) for accuracy. Inter‐rater agreement was *κ* = 0.99 and disagreements were discussed to reach a consensus.

### Risk of bias/quality assessment

We assessed risk of bias using the Revised Cochrane Risk‐of‐Bias tool for Randomised Trials (ROB‐2), developed by the Cochrane Collaboration. The ROB‐2 is a framework for evaluating the risk of material bias in randomised controlled trials. It categorises studies as either ‘low risk’, ‘some concerns’, or ‘high risk’ for bias across five domains which informs an overall risk of bias judgement. We assessed the following five domains: (1) bias arising from the randomisation process (selection bias); (2) bias due to deviations from intended interventions (selection bias); (3) bias due to missing outcome data (attrition bias); (4) bias in measurement of the outcome (detection bias); and (5) bias in selection of the reported result (reporting bias). Each study was assessed for risk of bias by one coder (EKD) and a random selection of 20% were coded by a second coder (EAM). Overall inter‐rater reliability was *κ* = 0.96, with 100% on domains one, two, four, five, and total risk of bias, and 75% agreement on domain three. Discrepancies on domain three were discussed to reach a consensus. All domains were assessed using information available in the published manuscripts. Judgements made for domain five were also based on information from trial registry searches and searches for published trial protocols.

### Risk of bias between studies (publication bias)

We assessed publication bias by visually inspecting the funnel plot for each outcome variable to determine whether asymmetry was present. Asymmetry was formally examined using Eggers tests of publication bias (Egger et al., 1997). We also used the Duval and Tweedie trim and fill procedure to estimate the overall effect size for each outcome after adjusting for publication bias.

### Meta‐analytic procedures

For each comparison between the CBM‐I training group versus a control group, the effect size indicating the difference between the two groups at post‐training and post‐stressor task was calculated for each outcome (Hedges' *g*) (Hedges & Olkin, 1985). Effect sizes were calculated by subtracting (at post‐training or post‐stressor) the average score of the CBM‐I training group from the average score of the control group and dividing the result by the pooled standard deviation. Because many studies had a small sample size, the effect size was corrected for small sample bias (Hedges & Olkin, 1985). Hedges *g* values were classified as small if they were 0.2, moderate if they were 0.5, and large if they were 0.8 (Cohen, 1988).

If a study had multiple CBM‐I experimental group types (e.g., a written training presentation and a spoken presentation), each group was included separately and compared to the relevant control group. If a study utilised multiple measures for the same outcome (e.g., both parent‐ and child‐reports of anxiety symptoms or multiple questionnaires used for anxiety symptoms), the mean of the effect sizes for each measure were calculated. The direction of the effect sizes was such that positive effect sizes indicated that the experimental group scored in the expected direction relative to the control group (e.g., the experimental group had higher positive bias, or lower anxiety).

All analyses were conducted in *R* version 4.3.1 using the *metafor* package (Viechtbauer, 2010). We expected high levels of heterogeneity among the studies, so we employed random effects meta‐analytic models in all analyses using restricted maximum likelihood estimators and Hartung‐Knapp‐Sidik‐Jonkman 95% confidence intervals (CIs). We also calculated the *I* ^2^ statistic and its 95% CI to estimate heterogeneity (Higgins et al., 2003). A value of 25%, 50% and above 75% were categorised as low, moderate and high heterogeneity respectively. We also calculated tau‐sqaured (*τ*2), which provides an estimate of the variability of the true effect size, and the prediction interval, which indicated the range of in which the true effect size of 95% of all populations will fall (Borenstein et al., 2017).

To examine the associations between effect size and moderators, we conducted multiple subgroup analyses (for categorical moderators) and mixed‐effect meta‐regressions (for continuous moderators) on the post‐training outcomes, using the same specifications previously outlined for the main effect models. Finally, we tested publication bias by inspecting the funnel plot on all outcome measures and by Duval and Tweedie's trim and fill procedure (Duval & Tweedie, 2000), which provides an effect size accounting for publication bias. We also conducted Egger's test of the intercept to determine the bias captured by the funnel plot and to test whether it was significant. Studies were categorised as outliers if there was no overlap between its effect size CI and the pooled effect size CI.

## RESULTS

### Selection and inclusion of studies

**FIGURE 1:**
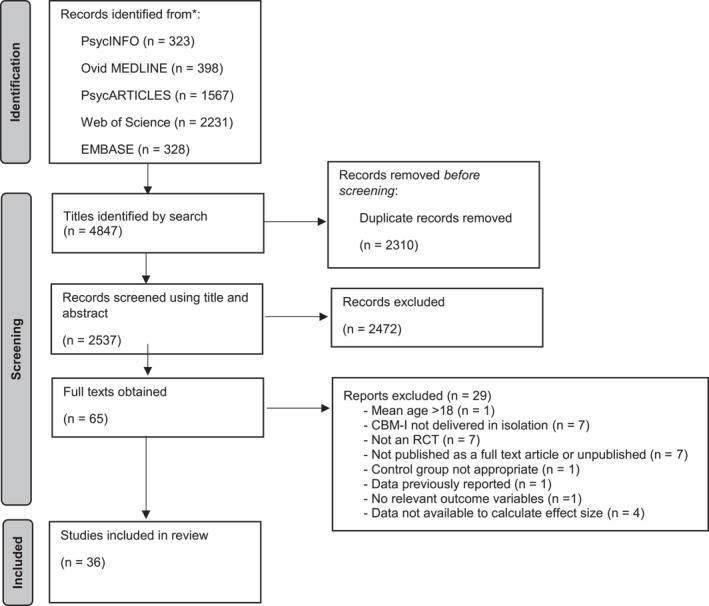
PRISMA diagram of selection of studies.

Following removal of 2310 duplicates, we screened the titles and abstracts of 2537 articles using the inclusion criteria of (a), (b) and (f). Sixty‐five articles were full text reviewed against the full eligibility criteria. A total of 36 studies were included in the meta‐analysis (see Figure 1).

### Study characteristics

Table 1 shows the study characteristics. This meta‐analysis includes 36 studies (40 experimental group comparisons) with data from 2692 participants. The study by Hiemstra et al. (2019) was treated as two studies, as it outlines two experimental procedures using different samples, leading to a total of 37 study samples included. Nine studies reported outcomes for anxiety symptoms post‐training, five studies reported outcomes for depression symptoms post‐training, 15 reported outcomes for state negative affect post‐training, and 11 studies reported outcomes for state negative affect following a stressor. In total, 25 studies reported outcomes for at least one measure of mental health (anxiety, depression or state negative affect). The majority of studies reported outcomes for either negative bias post‐training (*k* = 33) or positive bias (*k* = 23). Most studies (*k* = 23) were conducted with healthy or community samples, with eight studies using clinically diagnosed samples and six studies using samples with elevated mental health symptoms. Participants were aged between 6 and 19 years old, with 20 studies concerning children and 17 studies concerning adolescents. Of the total participants, 1385 (51.45%) were female. Nineteen studies compared CBM‐I training to neutral or mixed training, 10 studies compared CBM‐I training to negative training and eight studies compared CBM‐I to no training. The number of training sessions used ranged from 1 to 14, with 19 studies using a single‐session, nine studies using two to four sessions, and nine studies using more than five sessions.

**TABLE 1:** Study characteristics.

Study	Demographics (age group & % female)	Clinical status	Training paradigm	No. of training sessions	Control condition	Outcome measures					
						Anxiety post‐training	Depression post‐training	State negative affect post‐training	State negative affect post‐stressor	Negative interpretation bias post‐training	Positive interpretation bias post‐training
Belli and Lau (2014)	Adolescent	Healthy	Ambiguous situations (social)	1	Neutral training	NA	NA	VAS	NA	Recognition test	Recognition test
	79.7										
Bosmans et al. (2019)	Child	Healthy	Ambiguous situations (attachment)	1	Neutral training	NA	NA	NA	NA	NA	Recognition test
	66.7										
Burnett Heyes, et al. (2017)	Adolescent	Healthy	Mental imagery training (50% social)	2	Mixed imagery training	NA	NA	PANAS	NA	Recognition test	Recognition test
	0									Scrambled sentences task	Pleasantness ratings
Chan et al. (2015)	Adolescent	Healthy	Ambiguous situations	2	Neutral training	NA	NA	VAS	STAI‐S	Recognition test	Recognition test
	90.5										
De Voogd et al. (2017)	Adolescent	High anxiety or depression	Ambiguous situations	8	Neutral training (situations only)	SCARED	CDI	NA	VAS	Recognition test	NA
	73		Picture‐word training							Scrambled sentences task	
De Voogd et al. (2018)	Adolescent	Healthy	Ambiguous situations	8	Neutral training	SCARED	CDI	NA	VAS	Recognition test	NA
	60.7										
De Winter et al. (2017)	Child	Healthy	Ambiguous situations (attachment)	1	Neutral training	NA	NA	NA	NA	Recognition test	Recognition test
	55.1										
De Winter et al. (2018)	Child	Healthy	Ambiguous situations (attachment)	1	Neutral training	NA	NA	NA	NA	Recognition test	Recognition test
	54.8										
Fu et al. (2015)	Adolescent	Healthy	Ambiguous situations (50% social)	1	Mixed training	NA	NA	VAS	NA	Recognition test	Recognition test
	49										
Fu et al. (2013)	Adolescent	GAD or SAD	Ambiguous situations (50% social)	1	Mixed training	NA	NA	VAS	NA	IBQ	NA
	53.6									Recognition test	
Hiemstra et al. (2019) (study 1)	Child	Behaviour problem diagnosis	Ambiguous faces	5	Responses not reinforced	NA	NA	NA	NA	Ambiguous faces	NA
	0										
Hiemstra et al. (2019) (study 2)	Child	Behaviour problem diagnosis	Ambiguous faces	3	Responses not reinforced	NA	NA	NA	NA	Ambiguous faces	NA
	0										
Klein et al. (2015)	Child	Anxiety disorder	Ambiguous situations	14	Neutral training	SCAS‐C	NA	NA	NA	Ambiguous scenarios task	NA
	48.2					SCAS‐P					
Lau et al. (2013)	Adolescent	Healthy	Ambiguous situations	1	Negative training	NA	NA	VAS	VAS	Recognition test	Recognition test
	50										
Lau et al. (2011)	Adolescent	Healthy	Ambiguous situations	1	Negative training	NA	NA	VAS	NA	Recognition test	Recognition test
	64										
LeMoult et al. (2018)	Adolescent	MDD	Ambiguous situations	6	Neutral training	NA	CDI	NA	NA	Recognition test	Recognition test Blended words Scrambled sentences
	71.73–79.17						CDRS‐R			Blended words	
Lester et al. (2011b)	Child	Healthy	Ambiguous situations (animal & social)	1	Negative training	NA	NA	VAS	VAS	Ambiguous scenarios task	NA
	43.3										
Lester et al. (2011a)	Child	Healthy	Ambiguous situations (animal)	1	Negative training	VAS	NA	VAS	VAS	Ambiguous scenarios task	NA
	59.2										
Lothmann et al. (2011)	Adolescent	Healthy	Ambiguous situations	1	Negative training	NA	NA	VAS	NA	Recognition test	Recognition test
	53.7										
Muris et al. (2008)	Child	Healthy	Ambiguous situations	1	Negative training	NA	NA	NA	NA	Ambiguous scenarios task	NA
	51.4										
Nasiry et al. (2020)	Child	OCD	Ambiguous situations (OCD‐related)	8	Neutral training	OCI‐CV	NA	NA	NA	Ambiguous scenarios task	Ambiguous scenarios task
	54.29										
Orchard et al. (2017)	Child	SAD	Ambiguous situations (social)	3	No training	SCAS‐C	NA	NA	NA	Ambiguous scenarios task	Ambiguous scenarios task
	57					SCAS‐P					
Ren et al. (2021)	Adolescent	Healthy	Ambiguous situations (hostility related)	4	No training	NA	NA	NA	NA	Word sentence association paradigm	Word sentence association paradigm
	0									AIHQ	
Salemink and Wiers (2011)	Adolescent	Healthy	Ambiguous situations (social)	1	Neutral training	NA	NA	NA	NA	Recognition test	Recognition test
	53.5										
Schmidt and Vereenooghe (2021) (study 1)	Child	Healthy	Ambiguous situations	3	Neutral training	NA	NA	NA	NA	VASAPP	NA
	49.3		Ambiguous faces/body language								
Telman et al. (2013)	Adolescent	Healthy	Ambiguous situations	1	Negative training	NA	NA	VAS	NA	Recognition test	Recognition test
	78.3										
Van Bockstaele et al. (2020)	Adolescent	High aggression	Ambiguous scenarios (aggression related)	5	No training	NA	NA	NA	NA	Recognition test	Recognition test
	35.9										
Vassilopoulos et al. (2009)	Child	High social anxiety	Ambiguous situations (social)	3	No training	SASC‐R	CDI	NA	VAS	Ambiguous scenarios task	Ambiguous scenarios task
	81.3										
Vassilopoulos et al. (2014)	Child	High social anxiety	Ambiguous situations (social)	1	Negative training	NA	NA	VAS	VAS	Ambiguous scenarios task	Ambiguous scenarios task
	50										
Vassilopoulos and Brouzos (2016)	Child	Healthy	Ambiguous situations (social)	1	No training	SASC‐R	NA	NA	VAS	Ambiguous scenarios task	Ambiguous scenarios task
	47										
Vassilopoulos Brouzos et al. (2015)	Child	High aggression	Ambiguous situations (social)	3	No training	NA	NA	NA	NA	Ambiguous scenarios task	Ambiguous scenarios task
	11.8										
Vassilopoulos and Moberly (2013)	Child	Healthy	Ambiguous situations (social)	1	Negative training	NA	NA	VAS	NA	NA	NA
	57.4										
Vassilopoulos, Moberly et al. (2015)	Child	Healthy	Ambiguous situations (social)	1	Negative training	VAS	NA	VAS	VAS	Ambiguous scenarios task	Ambiguous scenarios task
	57.3										
Vassilopoulos et al. (2013)	Child	Healthy	Ambiguous situations (social)	3	No training	SASC‐R	CDI	NA	NA	NSECQ & PSEDQ	NSECQ & PSEDQ
	60.1										
White et al. (2016)	Child	High behavioural inhibition	Ambiguous situations	1	Neutral training	NA	NA	VAS	VAS	Ambiguous scenarios task	NA
	35.6										
Wolters et al. (2021)	Adolescent	OCD	Ambiguous situations (OCD‐related)	12	No training	C‐Y‐BOCS	NA	NA	NA	NA	NA
	46										
Zeng et al. (2023)	Adolescent	Healthy	Word sentence association paradigm	8	Neutral training	NA	NA	NA	NA	Word sentence association paradigm	NA
	53.7										

Abbreviations: AIHQ, Ambiguous intentions hostility questionnaire; CDI, Children's Depression Inventory; CDRS‐R, Children's Depression Rating Scale—Revised; C‐Y‐BOCS, Children's Yale‐Brown Obsessive Compulsive Scale; GAD, Generalized Anxiety Disorder; IBQ, Interpretation Bias Questionnaire; MDD, Major Depressive Disorder; NSECQ, Negative Social Events Catastrophization Questionnaire; OCD, Obsessive Compulsive Disorder; OCI‐CV, Obsessive Compulsive Inventory—Child Version; PANAS, Positive and Negative Affect Scale; PSEDQ, Positive Social Events Discounting Questionnaire; SAD, Social Anxiety Disorder; SASC‐R, Social Anxiety Scale for Children—Revised; SCARED, Screen for Child Anxiety Related Disorders; SCAS‐C/P, The Spence Children's Anxiety Scale—Child and Parent versions; STAI‐S, State‐Trait Anxiety Inventory—State Scale; VAS, Visual Analogue Scale; VASAPP, Vignette‐based Assessment of Social Ambiguity Processing in Pupils.

**FIGURE 2:**
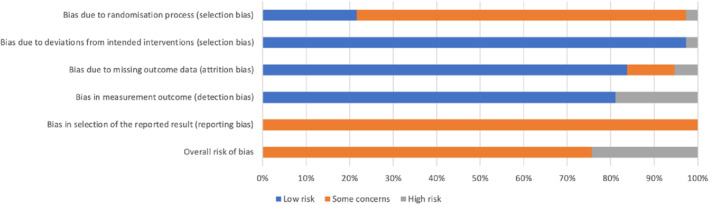
Summary of risk of bias across studies per criterion.

The risk of bias in many studies was considerable. The majority of studies (28/37) were rated as “some concerns”, nine studies were rated as “high risk”, and no studies were rated as “low risk” (see Figure 2 and Tables S1–S5).

### Overall effects of CBM‐I on mental health outcomes

#### Anxiety symptoms

**FIGURE 3:**
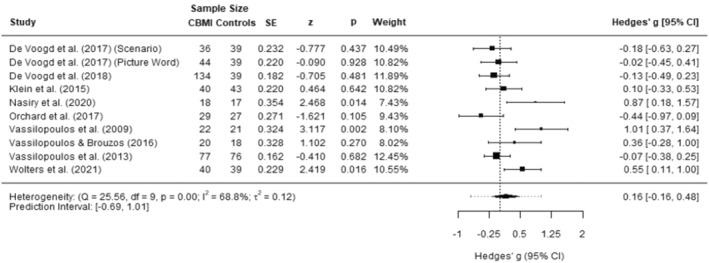
Forest plot of effect size of cognitive bias modification of interpretations (CBM‐I) versus control on anxiety symptoms.

Data from nine studies (10 experimental groups) were included. The effect size was *g* = 0.16 (95% CI −0.16–0.48), which corresponds to a small and nonsignificant effect. Heterogeneity was moderate (*I* ^2^ = 68.8%; 95% CI 31.63–92.48; *τ*2 = 0.12) and the prediction interval ranged from −0.69 to 1.01. The data of these analyses are presented in Figure 3.

There were no outliers identified, and we found no indication of publication bias. The Egger's test was not significant (*p* = 0.292) and no missing studies were identified using the Duval and Tweedie's trim and fill procedure. The funnel plot is presented in Figure S1.

#### Depressive symptoms

**FIGURE 4:**
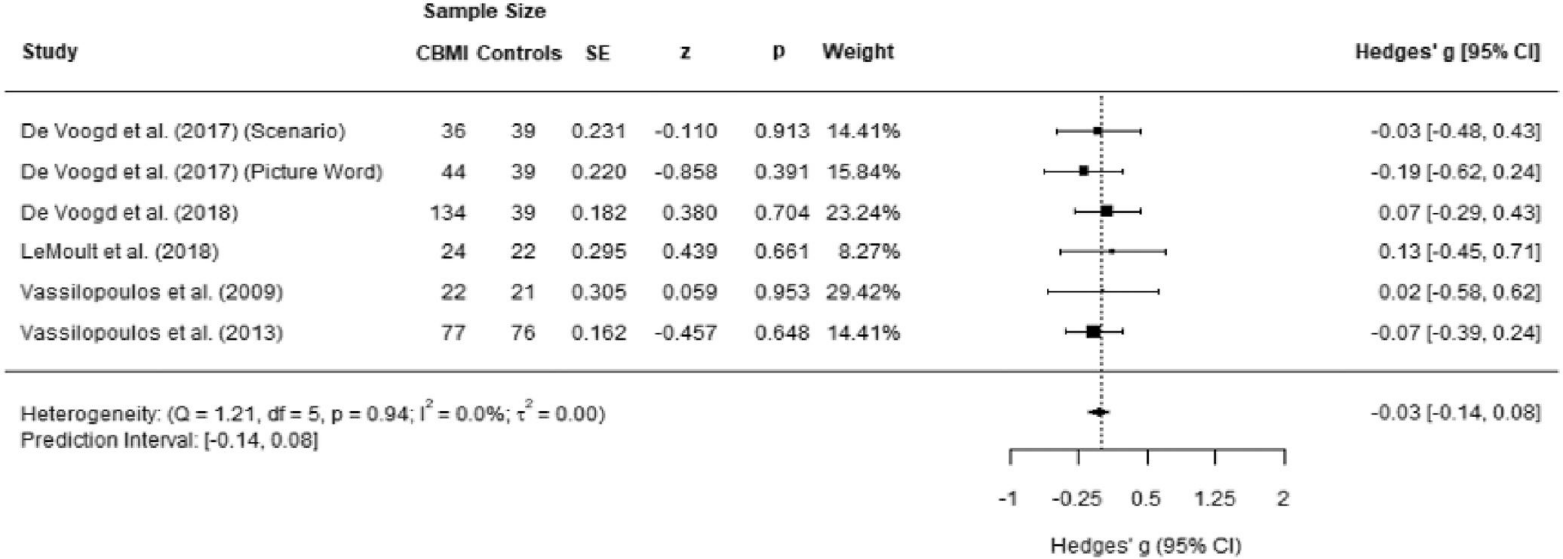
Forest plot of effect size of cognitive bias modification of interpretations (CBM‐I) versus control on depressive symptoms.

Data from five studies (6 experimental groups) were included. The effect size was *g* = −0.03 (95% CI −0.14–0.08), which was non‐significant. Heterogeneity was low (*I* ^2^ = 0.00%; 95% CI 0–30.95; *τ*2 = 0.00) and the prediction interval ranged from −0.14 to 0.08. The data of these analyses are presented in Figure 4.

There were no outliers identified and we found no indication of publication bias. The Egger's test was not significant (*p* = 0.568). One missing study was identified using the Duval and Tweedie's trim and fill procedure, and the updated effect size was *g* = −0.04 and remained non‐significant. The funnel plot is presented in Figure S2.

#### State negative affect

**FIGURE 5:**
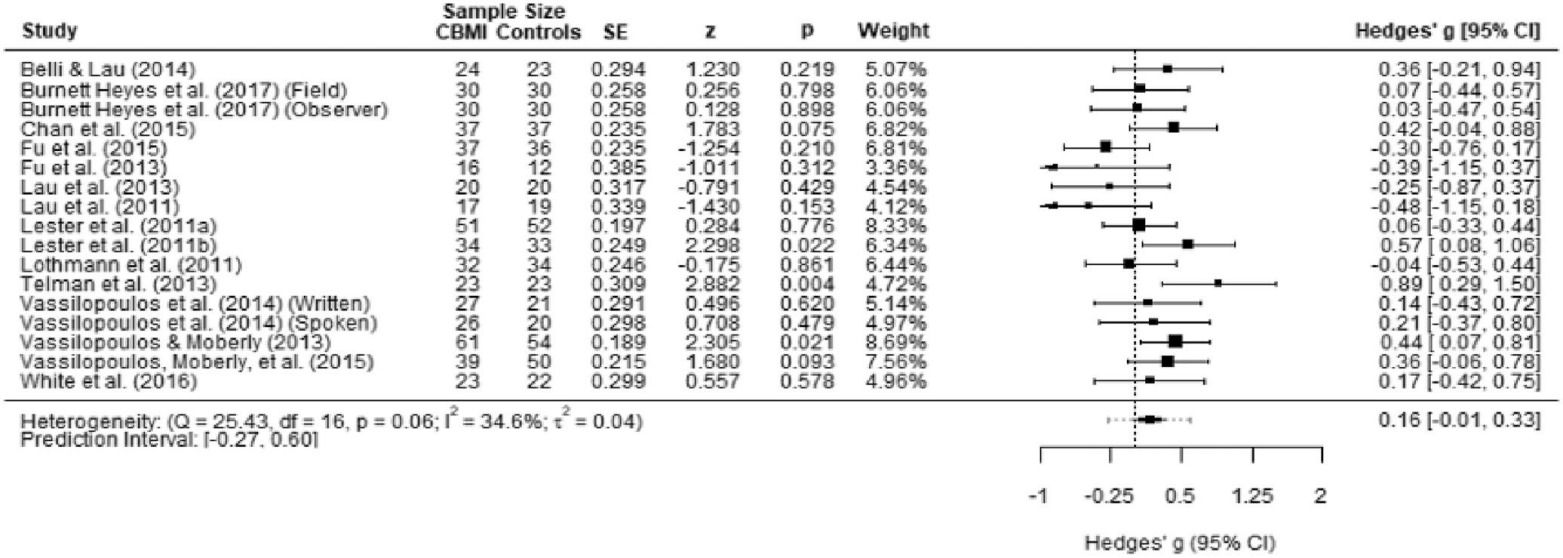
Forest plot of effect size of cognitive bias modification of interpretations (CBM‐I) versus control on state negative affect post‐training.

Data from 15 studies (17 experimental groups) were included. The effect size was *g* = 0.16 (95% CI −0.01–0.33), which corresponds to a non‐significant and small effect. Heterogeneity was low (*I* ^2^ = 34.6%; 95% CI 0–75.98; *τ*2 = 0.04) and the prediction interval ranged from −0.27 to 0.60. The data of these analyses are presented in Figure 5.

There were no outliers identified and we found no indication of publication bias. The Egger's test was not significant (*p* = 0.068). One missing study was identified using the Duval and Tweedie's trim and fill procedure, and the updated effect size was *g* = 0.19 which remained nonsignificant. The funnel plot is presented in Figure S3.

#### State negative affect post‐stressor

**FIGURE 6:**
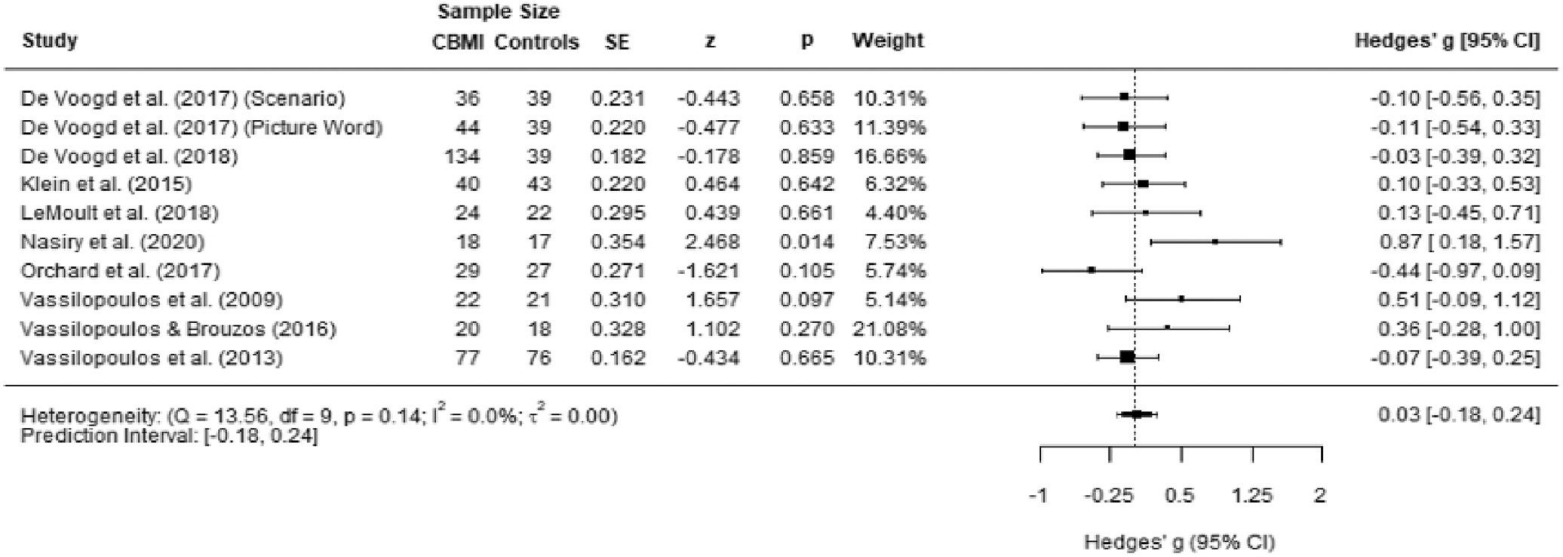
Forest plot of effect size of cognitive bias modification of interpretations (CBM‐I) versus control on state negative affect post‐stressor.

Data from 11 studies (13 experimental groups) were included. The effect size was *g* = 0.23 (95% CI −0.05–0.50), which was small and non‐significant. Heterogeneity was moderate (*I* ^2^ = 67.90%; 95% CI 36.68–89.46; *τ*2 = 0.13; 0.07) and the prediction interval ranged from −0.61 to 1.06. The data of these analyses are presented in Figure 6.

There were no outliers identified and we found no indication of publication bias. The Egger's test was not significant (*p* = 0.099) and no missing studies were identified using the Duval and Tweedie's trim and fill procedure. The funnel plot is presented in Figure S4.

### Overall effects on interpretation bias

#### Negative interpretation bias

Data from 33 studies (37 experimental groups) were included. The effect size was *g* = 0.78 (95% CI 0.48–1.08), which corresponds to a statisically significant moderate to large effect. Heterogeneity was high (*I* ^2^ = 90.6%; 95% CI 86.59–95.32; *τ*2 = 0.64) and the prediction interval ranged from −0.88 to 2.43. The data of these analyses are presented in Figure S5.

After removal of seven outliers (the CI of the effect size did not overlap with the pooled effect size CI), the effect size remained moderate and statistically significant (*g* = 0.59; 95% CI 0.47–0.72) and heterogeneity dropped significantly (*I* ^2^ = 41.9%; 95% CI 4.92–65.45). We found evidence of publication bias, as Egger's test was significant (*p* < 0.001). No missing studies were identified using the Duval and Tweedie's trim and fill procedure. The funnel plot is presented in Figure S6.

#### Positive interpretation bias

Data from 23 studies (25 experimental groups) were included. The effect size was *g* = 0.52 (95% CI 0.36–0.68), which corresponds to a statistically significant moderate effect. Heterogeneity was moderate (*I* ^2^ = 49.10%; 95% CI 18.73–78.06; *τ*2 = 0.07) and the prediction interval ranged from −0.04 to 1.08. The data of these analyses are presented in Figure S7.

After removal of 2 outliers (the CI of the effect size did not overlap with the pooled effect size CI), the effect size remained moderate and statistically significant (*g* = 0.50; 95% CI 0.37–0.63) and heterogeneity dropped significantly (*I* ^2^ = 22.3%; 95% CI 0–62.56). We found evidence of publication bias, as Egger's test was significant (*p* < 0.001). Seven missing studies were identified using the Duval and Tweedie's trim and fill procedure and the updated effect size was *g* = 0.36. The funnel plot is presented in Figure S8.

### Subgroup analyses

We conducted a series of subgroup analyses to examine outcomes of the moderator variables on all outcomes post‐training (Table S6). We did not find a statistically significant difference between groups for any categorical moderators (clinical status, age, control group type, or number of training sessions). Meta‐regressions also showed no statistically significant effect of age, gender, or number of training sessions (1–14) on any outcome measure (p's > 0.05, see Table S7).

## DISCUSSION

The aim of this meta‐analysis was to establish the efficacy of CBM‐I for children on psychometrically validated anxiety and depression symptom measures as well as measures of state negative affect. We also aimed to establish the extent to which CBM‐I reduces negative interpretations and increases positive interpretations in youth and the factors which might moderate the effects. This meta‐analysis updated previous meta‐analyses on the efficacy of CBM‐I in youth (Cristea et al., 2015; Krebs et al., 2017) by examining a greater number of studies and separating symptom and state outcomes pertaining to anxiety and depression.

Our results showed that CBM‐I had no significant effects on all mental health outcomes: anxiety symptoms, depressive symptoms and state negative affect. This contrasts with previous findings in youth which combined symptom and state measures of anxiety to find a small and significant effect (Krebs et al., 2017), but is consistent with findings by Cristea et al. (2015) which reported that CBM‐I had no significant effects on (mostly state) measures of general distress. Our results also showed that there were no significant effects of CBM‐I on negative affect following exposure to a stressor, which is inconsistent with Krebs et al.’s (2017) analysis which showed the effect on state measures is larger following a stressor. We note that according to our post‐hoc power analysis we should have been able to identify small effects with 31 studies with a mean sample size of 72 (the mean sample of included studies) with 80% power. Given we only had 10 comparisons for anxiety symptoms, 6 comparisons for depression symptoms and 17 comparisons for state negative affect, this indicates that our results may be due to low power. Alternatively, it indicates that CBM‐I has limited clinical utility for modifying mental health symptoms in youth. We discuss the implications of these results below.

Consistent with previous findings in youth (Cristea et al., 2015; Krebs et al., 2017) and adults (Cristea et al., 2015; Hallion & Ruscio, 2011), our results indicated that CBM‐I has a significant and moderate to large effect on reducing negative interpretation bias and a moderate effect on increasing positive interpretation bias. We included 12 additional comparisons for negative interpretation bias and seven additional comparisons compared to the most recent review (Krebs et al., 2017) indicating that this appears to be a consistent result. For positive interpretation bias only, the effect size decreased from moderate (*g* = 0.52) to small (*g* = 0.36) when we accounted for publication bias. The results for mental health outcomes and negative interpretation bias remained unchanged when we accounted for publication bias (if present) and outliers. Thus, CBM‐I remains a powerful and targeted way to influence interpretation bias outcomes in youth.

We found no moderating effect of any variable on any outcome. This is consistent with previous reviews on CBM‐I in youth which also did not find a moderating effect of age, gender, clinical status, and type of training group (Cristea et al., 2015; Krebs et al., 2017). These results are likely explained by the small number of studies in each of the subgroups, and so more studies are needed to fully evaluate these potential sources of heterogeneity across outcomes.

Overall, despite CBM‐I successfully modifying biases, our results indicate that this does not translate to youth mental health outcomes pertaining to anxiety and depression. This may indicate that CBM‐I has limited clinical utility in youth. However, before accepting this conclusion, a few issues warrant further discussion. First, the issue of heterogeneity across studies poses significant challenges to interpreting outcomes based on a synthesis of data. While attempts at evaluating these potential sources of heterogeneity were made in the current study, a lack of power to detect effects may have contributed to our non‐significant findings. Indeed, a disappointing meta‐analytic result may mask an effective CBM‐I intervention. For example, only one CBM‐I study has been conducted using a clinical child sample with multiple sessions (Klein et al., 2015) and despite positive outcomes on parent‐reported anxiety symptoms, this study has not been replicated. Most of the studies included in the current review included unselected analogue samples using a single‐session intervention to test causal mechanisms. This contrasts with CBM‐I research in adults which has been conducted more extensively on clinical or subclinical samples (e.g., Fodor et al., 2020) using multi‐session training. It seems likely that CBM‐I would have stronger effects in clinical samples where there are a greater number of sessions, but more studies are needed to confirm this.

Another source of heterogeneity is the type of control group used. While the use of a neutral or negative training condition makes sense to test specificity of intervention effects, it may not be suitable for assessing clinical efficacy, whereby small effects require large samples (Blackwell, 2020). The review by Fodor et al. (2020) on CBM‐I efficacy in adults with clinical or subclinical anxiety included 65 studies and found moderate effects compared to a waitlist control group and a small effect compared to sham (i.e., neutral) training. One solution to the issue of heterogeneity is the development of established protocols for evaluating multi‐session CBM‐I in RCTs involving youth clinical samples, with a comparison to a waitlist control. Additionally, replication of these studies is needed to extend the evidence base.

Second, CBM‐I only targets one mechanism associated with anxiety and depression, so it may be optimistic to expect it to improve symptoms. A more fruitful approach may be to combine CBM‐I with other therapeutic techniques, such as exposure or cognitive restructuring, and determine whether CBM‐I augments treatment outcomes. There is preliminary research in adults which suggests that CBM‐I may be a useful adjunct to CBT (Amir et al., 2015; Beard et al., 2019, 2021; Salemink et al., 2015; Schmidt et al., 2017; Williams et al., 2015). CBM‐I may also be a useful intervention to offer youth during a waitlist for conventional face‐to‐face CBT, overcoming issues of access. In youth samples, only one study has evaluated CBM‐I as a successful pre‐treatment/waitlist option for CBT (Wolters et al., 2021), thereby warranting further research.

Third, while there are many potential benefits of CBM‐I, such as its low cost and accessibility, participants often find it boring (de Voogd et al., 2017). Keeping individuals motivated and engaged to complete the training is vital to maximise its effects outside of laboratory settings. Interestingly, Cristea et al. (2015) found that CBM‐I had a small but stronger effect on mental health outcomes when they were delivered in school settings, perhaps due to increased adherence. Co‐designing digitally delivered CBM‐I interventions is one way to increase engagement and adherence in real‐world settings, which has been done successfully in CBM‐I trials on adults (Beard et al., 2021). In youth, different ways to improve engagement have been trialled, including tailoring content to individual concerns (e.g., Wolters et al., 2021), gamification (e.g., Salemink et al., 2022), and embedding the scenarios in a narrative context (e.g., Muris et al., 2009) However, further work is needed to evaluate the acceptability of these approaches and whether they improve treatment engagement.

This meta‐analysis has limitations. First, a lack of power to detect small effects on mental health outcomes was an issue for our analyses. Our aim to separate out outcomes for symptom and state measures led to fewer studies for each pooled outcome compared to if we had pooled all mental health outcomes together. However, we had a greater number of overall studies compared to previous reviews (Cristea et al., 2015; Krebs et al., 2017) and a comparable number of studies for negative affect outcome compared to Krebs et al.’s analysis. We encourage additional CBM‐I studies in youth that evaluate outcomes on anxiety and depressive symptoms using psychometrically robust measures to be conducted, so that our analyses can be replicated with sufficient power to detect a small effect. Second, most of the studies were assessed as having some risk of bias. This was largely due to insufficient information, particularly around the randomisation process and selective reporting of results. Future studies are encouraged to pre‐register trial protocols and provide sufficient details of pre‐specified analyses which will reduce the risk of bias. Third, we did not evaluate the efficacy of CBM‐I following a temporal lag (i.e., follow up). This was due to there being very few studies that included follow‐up assessments. It may be that it takes time for CBM‐I to have an impact on mental health outcomes, as this new style of processing information is consolidated, and so future studies are encouraged to include a follow up to determine the durability of effects.

## SUMMARY

This meta‐analysis is the first study to evaluate the efficacy of CBM‐I in children and adolescents on clinically relevant symptom measures of anxiety and depression, as well as broader measures of negative affect. Our results suggest that CBM‐I does not have a significant effect on any mental health outcome. Consistent with previous research, there was evidence that CBM‐I had a moderate to large effect on bias outcomes. Given the substantial level of variability across studies, there is a clear need to establish Randomized controlled trial protocols that evaluate CBM‐I in clinical or subclinical samples using validated symptom measures compared to a no training control group to determine its future as a clinical intervention. Ways to improve engagement in CBM‐I by tailoring content relevant to symptom domains and evaluating it in conjunction with other interventions may also be needed.

## AUTHOR CONTRIBUTIONS

**Gemma Sicouri**: Conceptualization; investigation; methodology; supervision; project administration; writing – original draft; writing – review & editing. **Emily Daniel**: Investigation; data curation; methodology; formal analysis; writing – original draft; writing – review & editing. **Michael Spoelma**: Investigation; data curation; methodology; formal analysis; writing – review & editing. **Elske Salemink**: Supervision; writing – review & editing. **Emma McDermott**: Methodology; writing – review & editing. **Jennie Hudson**: Conceptualisation; supervision, writing – review & editing.

## CONFLICT OF INTEREST STATEMENT

The authors have declared that they have no competing or potential conflicts of interest.

## ETHICAL CONSIDERATIONS

No ethical approval was required for this research review.

## Supporting information

Supplementary Information S1

## Data Availability

Data available on request from the authors.
