# Supplementary material for: Cognitive bias modification of interpretations for anxiety and depression in children and adolescents: A meta‐analysis
Source: JCPP Adv. 2023 Nov 16;4(1):e12207. doi: 10.1002/jcv2.12207 (PMC10933640; doi:10.1002/jcv2.12207)
Supplement: Supplementary file 1 — Supplementary Information S1 [file JCV2-4-e12207-s001.docx]

Online Supporting Information for: Cognitive Bias Modification of Interpretations for Anxiety and Depression in Children and Adolescents: A Meta-analysis and Systematic Review – by Sicouri et al.

Table S1: Risk of bias arising from the randomisation process

| **Risk of bias judgement** | **Number of studies** | **Explanation** |
| --- | --- | --- |
| Low risk | 8/37 | These studies provided sufficient information to determine that allocation was concealed until participants were enrolled and assigned. |
| Some concerns | 28/37 | These studies did not provide sufficient information to determine whether the allocation was concealed until participants were enrolled and assigned. |
| High risk | 1/37 | This study used a predictable allocation sequence (alternation), and it was likely that allocation sequence was not concealed due to the use of different coloured envelopes for each group. |

Table S2: Risk of bias due to deviations from the intended interventions

| **Risk of bias judgement** | **Number of studies** | **Explanation** |
| --- | --- | --- |
| Low risk | 36/37 | The CBM-I interventions in these studies were all guided by computers or flash cards with little personnel involvement during training. Considering this, it was seen as unlikely that deviations could arise, regardless of blinding status. |
| Some concerns | 0/38 | NA |
| High risk | 1/37 | This study excluded over 25% of participants randomized due to incomplete participation in the intervention. This makes it likely that there was a substantial impact on the results due to failure to analyze participants in the group in which they were randomized. |

Table S3: Risk of bias due to missing outcome data

| **Risk of bias judgement** | **Number of studies** | **Explanation** |
| --- | --- | --- |
| Low risk | 31/37 | These studies had less than 5% of the data missing or provided evidence that the result was not biased from missing data. |
| Some concerns | 4/37 | These studies had more than 5% of the data missing and dd not provide evidence that missingness did not bias the outcome. |
| High risk | 2/37 | These studies did not provide information on missing data, making it impossible to determine whether outcomes were impacted by missingness. |

Table S4: Risk of bias in measurement of the outcome

| **Risk of bias judgement** | **Number of studies** | **Explanation** |
| --- | --- | --- |
| Low risk | 30/37 | These studies all used appropriate measurement methods that were administered comparably to the intervention groups. Study participants were unlikely to be aware of their training group due to the use of neutral or negative training controls. |
| Some concerns | 0/37 | NA |
| High risk | 7/37 | These studies used no-training control groups, making it highly likely the participants were aware of their interventions and possible that this could have influenced outcomes. |

Table S5: Risk of bias in selection of the reported result

| **Risk of bias judgement** | **Number of studies** | **Explanation** |
| --- | --- | --- |
| Low risk | 0/38 | None of the identified studies were prospectively registered with clearly defined primary outcomes and analysis plans. |
| Some concerns | 37/37 | The majority of these studies were not prospectively registered in trial registration databases or trial protocol publications, making it impossible to determine whether selective reporting had occurred.  Four of the identified studies were prospectively registered on trial registration databases. For these studies, information was extracted on dates of registration and data collection, whether changes were made to the registry following the commencement of data collection, outcome measures for the primary outcome, measurement timepoints for the primary outcome, and analysis intentions. For three of these studies, the registrations did not specify outcome measures or analysis intentions for the primary outcome. For one of these studies, the registration specified the outcome measures but not the analysis intentions. |
| High risk | 0/38 | None of the identified studies demonstrated clear evidence (i.e., from a trial registration database or protocol publication) of selective reporting of multiple outcome measures or analyses. |

Table S6: Results of subgroup analyses

| **Moderator variable** | **Outcome variable** | **Subgroup** | **Hedge’s g (95% CI)** | **z** | ***p*** | ***I^2^*** | **Test for subgroup differences** | | |
| --- | --- | --- | --- | --- | --- | --- | --- | --- | --- |
|  |  |  |  |  |  |  | **Q** | ***df*** | ***p*** |
| Clinical status | Anxiety symptoms | Diagnosed/high symptom | 0.24 (-0.25, 0.73) | 1.19 | 0.280 | 75.34 | 0.45 | 1, 8 | 0.523 |
|  |  | Healthy | -0.04 (-0.50, 0.42) | -0.37 | 0.748 | 0.01 |  |  |  |
|  | Depressive symptoms | Diagnosed/high symptom | -0.04 (-0.26, 0.17) | -0.65 | 0.561 | 0.00 | 0.12 | 1, 4 | 0.747 |
|  |  | Healthy | -0.01 (-0.91, 0.89) | -0.15 | 0.905 | 0.00 |  |  |  |
|  | State negative affect | Diagnosed/high symptom | 0.08 (-0.31, 0.47) | 0.67 | 0.553 | 0.00 | 0.26 | 1, 15 | 0.618 |
|  |  | Healthy | 0.18 (-0.04, 0.40) | 1.77 | 0.101 | 47.21 |  |  |  |
|  | Negative bias | Diagnosed/high symptom | 0.65 (0.21, 1.08) | 3.20 | 0.006 | 86.09 | 0.48 | 1, 35 | 0.493 |
|  |  | Healthy | 0.87 (0.43, 1.31) | 4.13 | <0.001 | 92.94 |  |  |  |
|  | Positive bias | Diagnosed/high symptom | 0.53 (0.19, 0.87) | 3.72 | 0.007 | 40.89 | 0.01 | 1, 23 | 0.941 |
|  |  | Healthy | 0.52 (0.31, 0.73) | 5.25 | <0.001 | 55.43 |  |  |  |
| Age (categorical) | Anxiety symptoms | Adolescent | 0.05 (-0.48, 0.58) | 0.29 | 0.794 | 0.06 | 0.44 | 1, 8 | 0.525 |
|  |  | Child | 0.26 (-0.31, 0.84) | 1.18 | 0.291 | 76.33 |  |  |  |
|  | Depressive symptoms | Adolescent | -0.01 (-0.22, 0.20) | -0.15 | 0.893 | 0.00 | 0.21 | 1, 4 | 0.674 |
|  |  | Child | -0.05 (-0.54, 0.43) | -1.41 | 0.393 | 0.00 |  |  |  |
|  | State negative affect | Adolescent | 0.05 (-0.24, 0.34) | 0.37 | 0.721 | 49.72 | 2.28 | 1, 15 | 0.151 |
|  |  | Child | 0.29 (0.12, 0.47) | 4.05 | 0.007 | 0.00 |  |  |  |
|  | Negative bias | Adolescent | 0.55 (0.30, 0.80) | 4.67 | <0.001 | 71.46 | 1.85 | 1, 35 | 0.183 |
|  |  | Child | 0.99 (0.43, 1.55) | 3.71 | 0.002 | 94.25 |  |  |  |
|  | Positive bias | Adolescent | 0.55 (0.30, 0.79) | 4.88 | <0.001 | 49.30 | 0.12 | 1, 23 | 0.734 |
|  |  | Child | 0.49 (0.24, 0.75) | 4.21 | 0.001 | 53.36 |  |  |  |
| Control group type | Anxiety symptoms | Negative training | - | - | - | - | 0.32 | 1, 8 | 0.586 |
|  |  | Neutral training | 0.03 (-0.38, 0.44) | 0.21 | 0.842 | 23.55 |  |  |  |
|  |  | No training | 0.26 (-0.42, 0.94) | 1.06 | 0.350 | 78.66 |  |  |  |
|  | Depressive symptoms | Negative training | - | - | - | - | 0.21 | 1, 4 | 0.674 |
|  |  | Neutral training | -0.01 (-0.22, 0.20) | -0.15 | 0.893 | 0.00 |  |  |  |
|  |  | No training | -0.05 (-0.54, 0.43) | -1.41 | 0.393 | 0.00 |  |  |  |
|  | State negative affect | Negative training | 0.21 (-0.05, 0.48) | 1.84 | 0.100 | 44.71 | 0.74 | 1, 15 | 0.402 |
|  |  | Neutral training | 0.07 (-0.20, 0.35) | 0.66 | 0.536 | 18.94 |  |  |  |
|  |  | No training | - | - | - | - |  |  |  |
|  | Negative bias | Negative training | 1.35 (0.35, 2.35) | 3.06 | 0.014 | 95.44 | 2.47 | 2, 34 | 0.100 |
|  |  | Neutral training | 0.58 (0.26, 0.90) | 3.76 | 0.001 | 86.24 |  |  |  |
|  |  | No training | 0.54 (0.35, 0.72) | 6.92 | <0.001 | 0.00 |  |  |  |
|  | Positive bias | Negative training | 0.80 (0.37, 1.24) | 4.51 | 0.004 | 60.95 | 2.08 | 2, 22 | 0.149 |
|  |  | Neutral training | 0.42 (0.21, 0.64) | 4.43 | 0.001 | 32.69 |  |  |  |
|  |  | No training | 0.42 (0.07, 0.76) | 2.97 | 0.025 | 37.29 |  |  |  |
| Number of sessions (categorical) | Anxiety symptoms | Single session | 0.36 (-0.28, 1.00) | 1.10 | 0.270 | - | 0.07 | 2, 7 | 0.930 |
|  |  | 2-4 sessions | 0.14 (-1.68, 1.97) | 0.34 | 0.765 | 0.002 |  |  |  |
|  |  | 5+ sessions | 0.15 (-0.26, 0.55) | 0.94 | 0.392 | 59.05 |  |  |  |
|  | Depressive symptoms | Single session | - | - | - | - | 0.21 | 1, 4 | 0.674 |
|  |  | 2-4 sessions | -0.05 (-0.54, 0.43) | -1.41 | 0.393 | 0.00 |  |  |  |
|  |  | 5+ sessions | -0.01 (-0.22, 0.20) | -0.15 | 0.893 | 0.00 |  |  |  |
|  | State negative affect | Single session | 0.15 (-0.06, 0.36) | 1.53 | 0.150 | 44.63 | 0.02 | 1, 15 | 0.896 |
|  |  | 2-4 sessions | 0.19 (-0.36, 0.73) | 1.49 | 0.276 | 0.00 |  |  |  |
|  |  | 5+ sessions | - | - | - | - |  |  |  |
|  | Negative bias | Single session | 1.01 (0.48, 1.54) | 4.02 | 0.001 | 92.43 | 1.18 | 2, 34 | 0.320 |
|  |  | 2-4 sessions | 0.67 (0.14, 1.20) | 2.83 | 0.018 | 88.40 |  |  |  |
|  |  | 5+ sessions | 0.43 (-0.08, 0.94) | 2.00 | 0.086 | 83.67 |  |  |  |
|  | Positive bias | Single session | 0.64 (0.41, 0.88) | 5.82 | <0.001 | 54.41 | 1.52 | 2, 22 | 0.242 |
|  |  | 2-4 sessions | 0.35 (0.08, 0.63) | 2.96 | 0.018 | 40.68 |  |  |  |
|  |  | 5+ sessions | 0.43 (-1.26, 2.13) | 3.25 | 0.190 | 0.00 |  |  |  |

Table S7: Results of meta-regression analyses

| **Moderator variable** | **Outcome variable** | **Regression coefficient (95% CIs)** | ***p*** | ***I^2^*** |
| --- | --- | --- | --- | --- |
| Age (continuous) | Anxiety symptoms | -0.0500 (-0.1857, 0.0857) | 0.420 | 70.57 |
|  | Depressive symptoms | 0.0023 (-0.0600, 0.0646) | 0.923 | 0.00 |
|  | State negative affect | -0.0481 (-0.1192, 0.0229) | 0.167 | 14.53 |
|  | Negative bias | -0.0747 (-0.2077, 0.0583) | 0.261 | 91.18 |
|  | Positive bias | 0.0046 (-0.0607, 0.0698) | 0.886 | 41.05 |
| Gender (% female) | Anxiety symptoms | 0.0053 (-0.0319, 0.0425) | 0.750 | 73.27 |
|  | Depressive symptoms | 0.0052 (-0.0137, 0.0242) | 0.485 | 0.00 |
|  | State negative affect | 0.0043 (-0.0031, 0.0117) | 0.231 | 31.01 |
|  | Negative bias | -0.0077 (-0.0205, 0.0050) | 0.225 | 90.35 |
|  | Positive bias | -0.0020 (-0.0088, 0.0049) | 0.554 | 50.58 |
| Number of sessions (continuous) | Anxiety symptoms | 0.0053 (-0.0821, 0.0927) | 0.892 | 72.48 |
|  | Depressive symptoms | 0.0043 (-0.0522, 0.0609) | 0.841 | 0.00 |
|  | State negative affect | 0.0288 (-0.4321, 0.4898) | 0.896 | 39.25 |
|  | Negative bias | -0.0772 (-0.1730, 0.0186) | 0.111 | 89.89 |
|  | Positive bias | -0.0365 (-0.1371, 0.0641) | 0.460 | 50.73 |


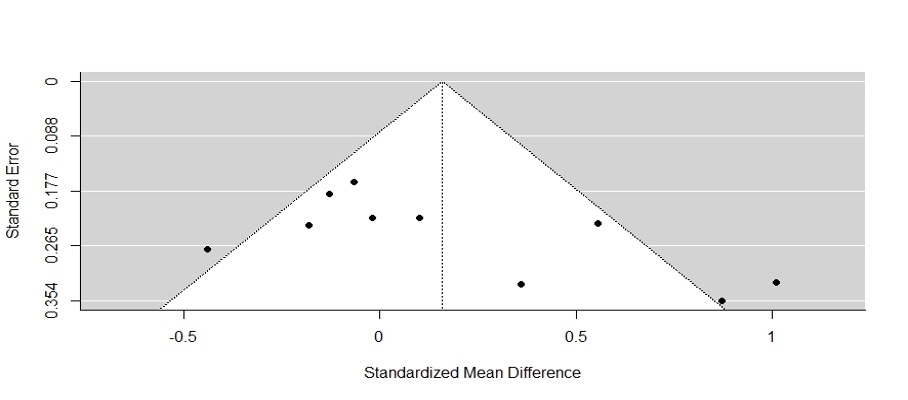
Figure S1: Funnel plot of publication bias for anxiety symptoms

Figure S2: Funnel plot of publication bias for depressive symptoms


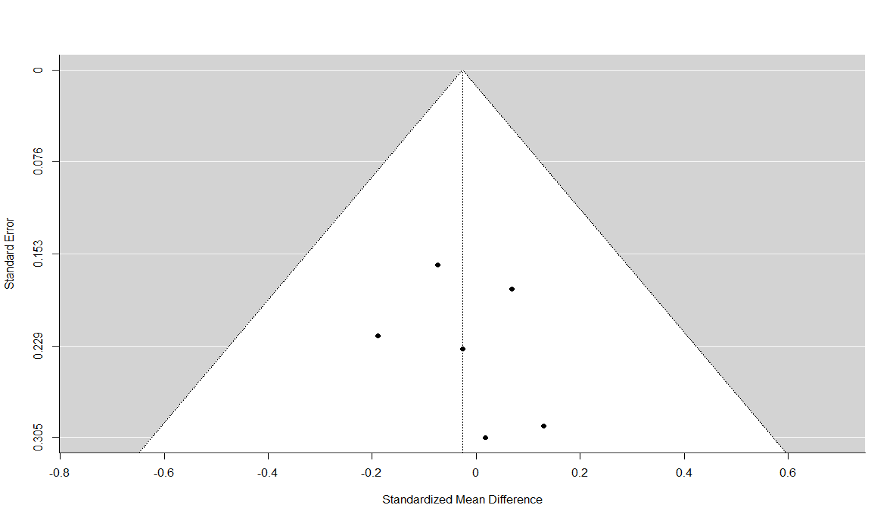


Figure S3: Funnel plot of publication bias for state negative affect post-training


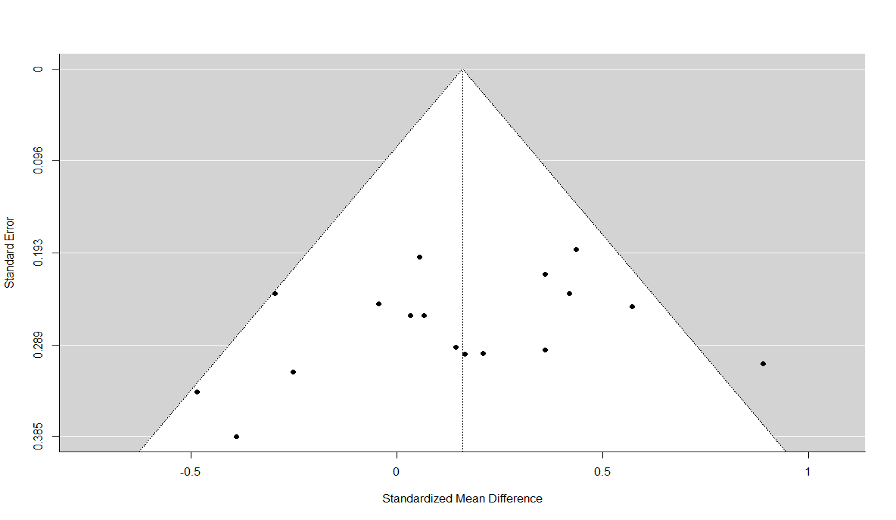


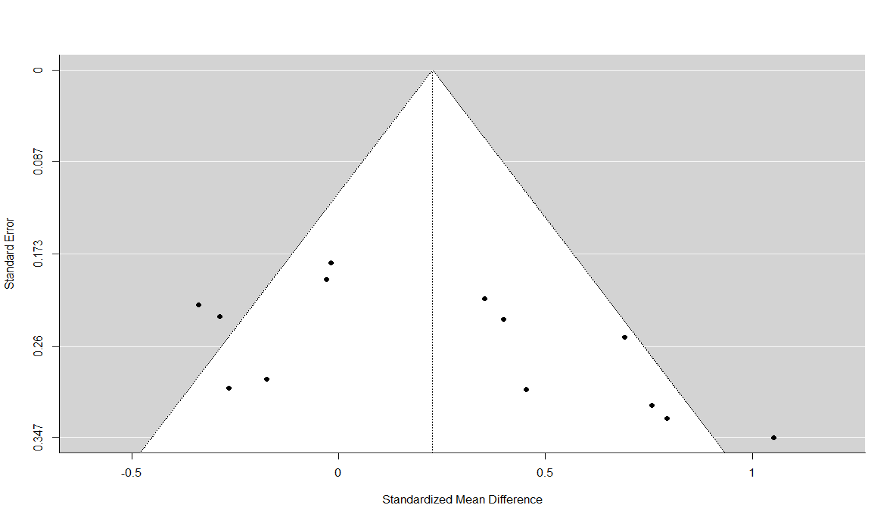
Figure S4: Funnel plot of publication bias for state negative affect post-stressor


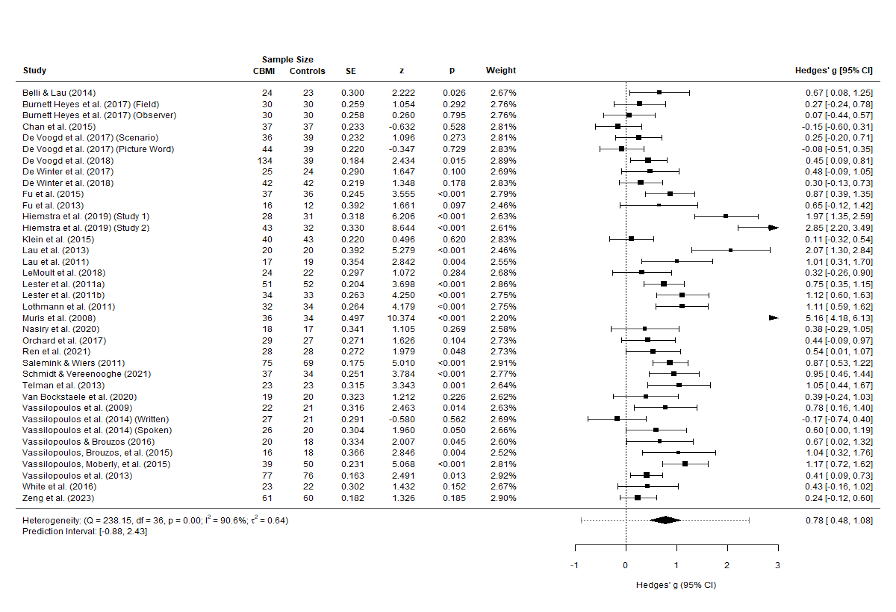
Figure S5: Forest plot of the effect size of CBM-I versus control on negative interpretation bias


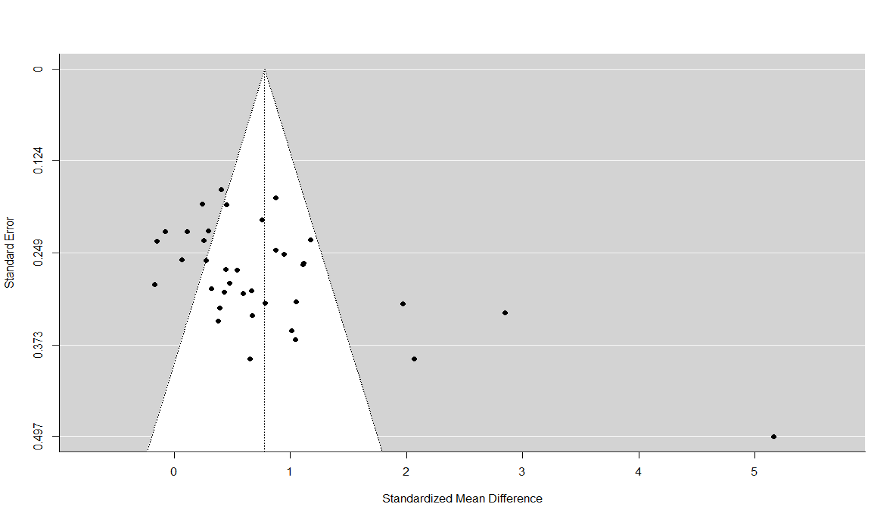
Figure S6: Funnel plot of publication bias for negative interpretation bias


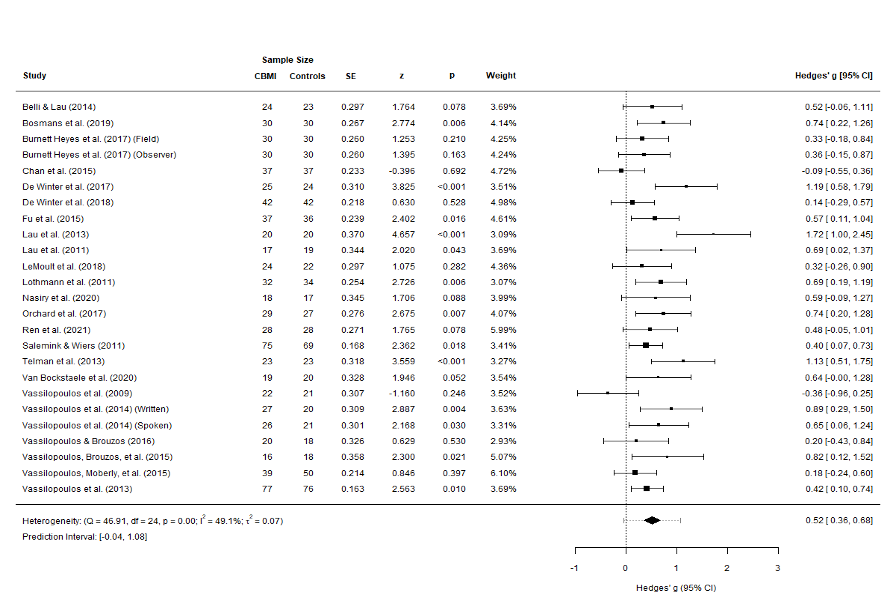
Figure S7: Forest plot of the effect size of CBM-I versus control on positive interpretation bias


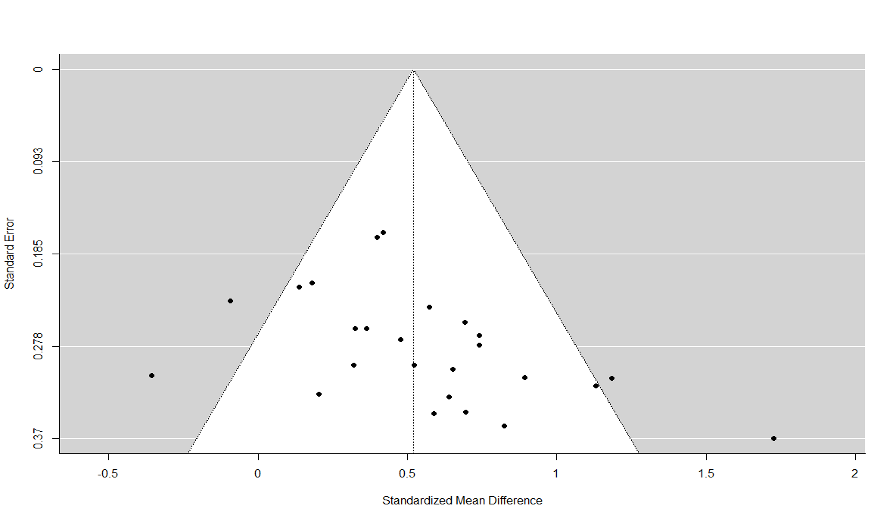
Figure S8: Funnel plot of publication bias for positive interpretation bias
